# A Case of Congenital Strangulated Hernia

**Published:** 1867-07

**Authors:** John Root

**Affiliations:** Batavia


ART. II.—*A case of Congenital Strangulated Hernia.* BY JOHN ROOT, M. D.

I report the following case of strangulated congenital hernia as possessing no particular interest, only in respect to the age of the little patient. The following paragraph is taken from *Cooper's Surgical Dictionary*, article "Hernia:"

"If the subject be an infant, the case is not often attended with much difficulty or hazard, the reduction being easy as well as the descent, and though from neglect or inattention the bowel may fall down again, yet it is easily replaced and mischief seldom produced; Mr. Pott says seldom, because he has seen an infant one year old die of a strangulated hernia which had not been down two days, with all the symptoms of mortified intestine."

Monday, June 24, 1867, was called to see a child of German parents, two years old, under the care of Dr. A. P. Jackson, of Cary. The child had a congenital scrotal hernia, left side, which had become irreducible the Thursday previous. Dr. Jackson was called Friday morning, and found so much inflammation that he directed his attention chiefly to subduing this without attempting reduction. Saturday and Sunday the same plan was continued. Monday morning the Doctor put the patient under the influence of a mixture of ether and chloroform, and attempted to reduce the hernia, but without success.

The same day at 4 P. M. I first saw the patient with Dr. J. There were the usual symptoms of strangulated hernia—vomiting, constipation, abdominal tenderness, frequent pulse, etc. The hernial tumor was quite large, and the lower half of it of a deep red color. The child being rendered insensible by the mixture formerly given, reduction was attempted by taxis, after having for some time thrown the spray of ether on the tumor. The hips were elevated and head depressed. Attempts at reduction were continued for two hours, but with no success, and we reluctantly came to the conclusion that the only relief was in an operation. This course was pursued. The patient still under chloroform, I dissected to the hernial sac, when about an ounce and a half or two ounces of serum escaped. Only one small artery required the ligature. The strangulation was found to be complete, and this rendered the division of the stricture rather difficult. The reduction was accomplished and the wound dressed in the usual manner. The recovery is complete. During the operation the child became convulsive, with irregular respirations, during which, of coure, all progress was suspended. This was probably caused by the anæsthetic.

Batavia, July 5th, 1867.

---

The London Surgical Home and the Operation of Clitoridectomy.—Mr. Baker Brown and Mr. Philip Harper, state that, *solely* in deference to the opinion of the medical press on the subject of clitoridectomy, they have determined not to perform the operation in this institution pending professional inquiry into its validity as a scientific and justifiable operation.—*Lancet.*

